# Cost Effectiveness of Mobile versus Fixed Computed Tomography and Magnetic Resonance Imaging: A Systematic Review

**Published:** 2019-08

**Authors:** Marita MOHAMMADSHAHI, Minoo ALIPOURI SAKHA, Atefeh ESFANDIARI, Maryam SHIRVANI, Ali AKBARI SARI

**Affiliations:** 1. Department of Health Management and Economics, School of Public Health, Tehran University of Medical Sciences, Tehran, Iran; 2. Department of Health Policy, School of Medicine, Bushehr University of Medical Sciences, Bushehr, Iran

**Keywords:** Computed tomography (CT), Magnetic resonance imaging (MRI), Mobile technology, Health technology assessment

## Abstract

**Background:**

Mobile technologies are widely used in healthcare. The purpose of this study was to compare the effectiveness and cost-effectiveness of fixed computed tomography (CT) and magnetic resonance imaging (MRI) with the mobile ones.

**Methods:**

In this systematic review, PubMed, Cochrane Library, Scopus and CRD database were searched from 1995 to 2015. The data on safety and effectiveness of technologies were extracted from included studies. Because the review showed no significant differences in the performance of mobile CT and MRI compared to the fixed ones, then a cost minimization approach was used to explore the cost-effectiveness of three scenarios.

**Results:**

Twenty two articles were included in the review that showed no statistically significant differences in the performance of mobile MRI and CT scan compared to the fixed ones. The cost minimization approach showed that the third scenario based on purchasing a common mobile MRI and CT scan; and using it by two or more hospitals that are in rational distance from each other is associated with the lowest costs, so it is the most cost-effectiveness strategy.

**Conclusion:**

The performance of Mobile CT and mobile MRI is comparable to the fixed ones; and using a combined mobile CT and MRI by two or three hospitals is the most cost-effective approach.

## Introduction

The Computed Tomography (CT or CAT scan) and Magnetic Resonance Imaging (MRI) are diagnostic imaging modalities that produce slice cross-sectional images of various anatomical organs (1, 2). A CT scanner uses a narrow x-ray tube located directly opposite to a digital x-ray detector and rotates around a patient’s body by a circular opening of a donut-shaped structure (1, 3). The MRI combines a powerful magnetic field with an advanced computer system. Because it does not have the risk of radiation, it causes little harm compared to other imaging modalities that utilize x-ray technology like CT scan and radiography.

The use of mobile CT and MRI scanners have been increasing over time in many countries due to improvements in social, economic, and technological factors. The mobile CT and MRI scanners can be placed on tyre or trailer to move within hospitals or from one area to another. The CT is one of the first technologies that availed in a movable form and the emergence of movable MRI is believed largely due to the success of the movable CT (4, 5). The indications of mobile scanners are similar to the fixed scanners; for example they can use for diagnostic purpose (tomur, truma, Unexplained chronic headache and seizure(s) and etc.), staging of the diseases (cancers, tumors, and etc.), screening and follow up the trend of diseases (6).

The diagnostic imaging services were formerly provided to individuals using the CT and MRI within the placement of the scanner. However, with the advancement of technology, the mobile modalities became closer to the patients. The demands for the mobile MRI and CT-Scan scan are mainly influenced by the costs and waiting list of the patients. In Canada, the purchasing of the mobile CT-Scan and MRI devices was attractive from the point of patients because it saves the costs of travel and about $ 118 per scan per patient. The cost of the initial investment in the fixed MRI and CT-Scan was higher than that of the mobile scanners (7, 8). The installation and establishment of the fixed MRI and CT-scan require the preparation of a building. In addition, specific requirements are needed for the designing of each room. Despite the mobile MRI and CT-Scan require less initial investment cost, purchasing them can be more expensive than renting from a private company. Although CT-scan and MRI technology have high executory costs these costs will be compensated with increased the results, flexibility and other costs (especially indirect costs such as travel costs for patients that do not have access to these services) (9). Outcomes and effectiveness of mobile CT-Scan and MRI do not have differ from fixed CT-Scan and MRI. But in general, positive effects such as the reduction staff workload, reduction in the time of receiving the services, reduction in the length of stay in the hospital, managing the patients more accurately and increased access to patients are more than negative effects (10–13).

CT-Scan and MRI machines are considered very valuable in diagnosis because they are noninvasive. However special caution should be taken when using them. Since in CT-scan (fixed or mobile) patient is exposed to radiation, attention to the required dose is necessary (14, 15). Moreover, as magnetic field attracts iron objects, in MRI, it may cause potential harm to patients and anybody standing in the direction of these objects. For caution, it is better to use MRI if necessary and with physicians prescription. Moreover, food and Drug Administration (FDA) confirmed the efficacy and safety of the mobile CT-Scan and MRI devices (14).

Since no study in this field has been done in Iran, this study aimed to compare fixed versus mobile CT and MRI scanners. The findings are believed to contribute to the decision in choosing the appropriate technology for use in the context of Iran.

## Materials and Methods

We used a comparative cost-effectiveness analysis between mobile and fixed CT and MRI scanners. This was based on a systematic review of published literature. In review all the articles which were published in English from Jan 1995 to Dec 2015 were searched (Table 1). Experimental (randomized clinical trials, quasi-randomized trials, non-randomized trials), Quasi-experimental, observational (cohort), systematic reviews, health technology assessment and economic evaluation study designs were eligible for the analysis. Furthermore, two co-authors independently assessed the eligibility of the retrieved records starting from the title, and then the abstract, and the content of the records. The potentially eligible records were first identified and further assessed for the inclusion in the analysis. The full texts of the records that fulfilled the PICO criteria were further scrutinized (considering patients who were scanned with mobile CT and MRI scanners as the population, the diagnostic imaging as intervention, the patients scanned with the fix CT and MRI as comparison groups, and the diagnostic power, quality, safety and costs of mobile CT and MRI as outcomes).

**Table 1:** Search strategy of databases

***Table :Search strategy***	***Databases***
Cochrane Library	((Magnetic Resonance Imaging OR MRI OR (Magnetic NEAR/2 Resonance NEAR/2 Imaging) OR MRI) OR (Computed NEAR/2 tomography NEAR/2 OR CT-scan AND mobile AND portable)
PubMed	((Magnetic Resonance Imaging OR MRI OR (Magnetic Resonance Imaging^*^) OR MRI) OR (Computed tomography^*^ OR CT-scan ) AND mobile^*^ AND portable^*^)
SCOPUS	((Magnetic Resonance Imaging OR MRI OR (Magnetic Resonance Imaging) OR MRI) OR (Computed tomography OR CT-scan) AND mobile AND portable)
CRD	((Magnetic Resonance Imaging OR MRI OR (Magnetic Resonance Imaging) OR MRI) OR (Computed tomography OR CT-scan) AND mobile AND portable)

**Fig. 1:**
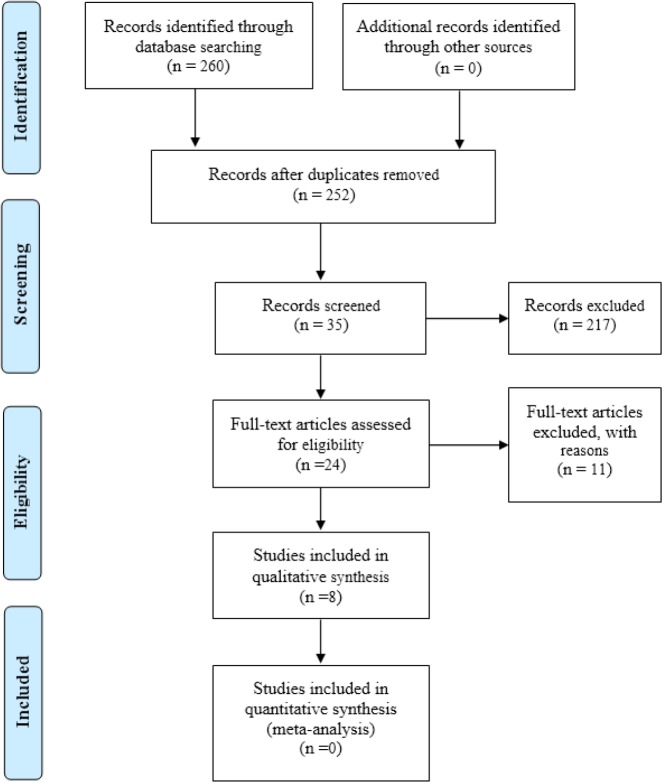
PRISMA diagram according to CASP criteria

The quality of each study was also assessed using the Critical Appraisal Skills Program (CASP) criteria. Figure 1 shows the schematic presentation of the selection process of articles.

Outcomes*:* An economic evaluation that focused on the effectiveness of the scanners. The sensitivity, specificity, quality of images (Pixel, Voxel) and accessibility to the scanners were outcomes of the study. The technologies were divided into those that were mobile between and within hospitals (also called portable scanners). Since the purpose of this study was to investigate mobile technology between hospitals, accordingly, eight (six on CT scan, one on MRI and the remaining one on both CT and MRI) studies in systematic review were eligible.

### Cost data:

The costs of the mobile and fixed CT and MRI scanners included both the current and capital expenditures. The accurate cost estimates also consider other costs such as the costs of travel to receive the imaging services, costs of work absenteeism (both the patients and their family members), the cost of meals and accommodation. The average cost of both the mobile and fixed scanners include the labor wages, consumption costs, operating costs including repairing and maintenance, overhead costs and the purchasing costs by considering the maximum use of the scanner efficiently over a full year assuming the scanner works four shifts per day. The costs of both the mobile and fixed scanners from hospital perspective are summarized in Table 2.

**Table 2:** Fixed and variable costs for mobile and fixed MRI and CT Scan

***Costs***	***Fixed MRI & CT Scan***	***Mobile MRI & CT Scan***		
Applicable model	-	Ownership model	Renting model	
Type of services	-	-	Full services like staff, Facilities Maintenance, …	Partial services
Fixed costs	Equipment Building, staff	Equipment, Mobile Trailer and Tractor, Facilities Maintenance, staff	Based on pay for day: all the costs are semi-variable	Based on pay for day: staff + semi-variable costs
Variable costs	Operating Supplies, Equipment Upgrades, Facilities Maintenance	Van Driver & Gasoline, Operating Supplies, Facilities Maintenance	Service Contract, Van Driver & Gasoline, Equipment Upgrades, Operating Supplies, Facilities Maintenance	Service Contract, Van Driver & Gasoline, Equipment Upgrades, Operating Supplies, Facilities Maintenance
Indirect costs	Time and travel costs of patients	Time and travel costs of patients	Time and travel costs of patients	Time and travel costs of patients

The information was obtained through online conversations and phone calls to companies producing mobile MRI and CT scan, domestic and foreign companies, and the Department of Medical Devices at the Ministry of Health and Medical Education (MOHME) of Iran. The cost of repairing and maintenance services of the devices were obtained from the Engineering Unit of the MOHME and by calculating the estimated cost of repairing and maintenance by the service provider companies. However, it was difficult to estimate the cost of repairing and maintenance of the mobile scanners, because these technologies are not in use in the country. Hence, 15% of the annual depreciation expense was considered as the repairing and maintenance costs. By interviewing an importing company, the service life of the devices was estimated at 10 yr. Besides article 151 of the Direct Tax Law of 1980, the depreciation schedule for hospital equipment is set at 10 yr (16).

### Economic evaluation:

Because the review showed no significant differences in the performance of mobile CT and MRI compared to the fixed ones, then a cost minimization approach was used to explore the cost-effectiveness. Thus, we designed different scenarios and in each scenario, three hospitals in three cities that were close to each other (max distance 75 km) and about 6,000 and 4800 patients use CT and MRI respectively each year. Costs related to a patient was calculated according to the lost working hours. It was based on the minimum basic salary of a worker (204 $) in Iran announced by the Ministry of Labor and Social Welfare (17). This study considered three scenarios. The first scenario was about purchasing and establishing one fixed MRI or CT machine in a hospital located in a city and serves as a referral hospital to other hospitals of two other cities located at about 75 km distance. Thus, the patients from the two other cities can use only this referral center for the imaging whenever they are requested to get the diagnostic service (Assuming a maximum 75 km distance to the central hospital and referring the patients with a relative). The second scenario was purchasing and establishing one fixed MRI or CT machine in the hospitals of the three cities that lacked these fixed devices (Assuming a maximum 75 km distance and referring the patients with a relative). The third scenario was purchasing and establishing one mobile MRI or CT machine in the hospitals of the three cities that lacked the fixed devices (Assuming a maximum 75 km distance and referring the patients with a relative). The three scenarios assumed that the technologies provided services to the referred patients and their relatives.

### Sensitivity Analyses:

According to the method which used in cost-effectiveness analyses, the sensitivity analysis was done for the common costs for each scanner.

### Safety:

The safeties of the devices were analyzed by reviewing the records of the final review, from observational studies and from the FDA site.

## Results

The literature search yielded a total of 24 records that fulfilled the eligibility criteria (Table 1), of which 17 articles were on CT, six were on MRI and the remaining one was on both CT and MRI. Further, three of the studies were on CT and MRI Health Technology Assessment (HTA), four were systematic reviews, 14 clinical trial studies and the rest one was a comparative study of mobile and fixed CT and MRI. But only 8 studies reported on the mobile scanners (six on CT scan, one on MRI and the remaining one on both CT and MRI) (Table 3). In none of these records was a measure such as odds ratio, relative risk and probability reported for comparision the effectiveness of mobile versus fixed MRI and CT.

**Table 3:** Studies of systematic review about the mobile scanners

***Title***	***Authors***	***Year***
A comparison of fixed and mobile CT and MRI scanners	Janis Reeve, et al	1995
HTA of Magnetic Resonance Imaging	Gillet Pierre and et.al	2006
Computed Tomography Scanners for Patients in Rural or Remote Locations: Clinical and Cost-Effectiveness	CADTH (Canadian Agency for Drug and Technologies in Health)	2014
Mobile computed tomography: prehospital diagnosis and treatment of stroke	Ebinger M, et al	2015
Evaluation of head examinations produced with a mobile CT unit	Matson MB, et.al	1999
Diagnosis and treatment of patients with stroke in a mobile stroke unit versus in hospital: a randomized controlled trial	Walter Silke, et.al	2012
Mobile Computed Tomography Scanner for Head and Neck Imaging	Tsang Kwai-fan Ice, et.al	2009
The Mobile Hospital Technology Industry: Focus on the Computerized Tomography Scanner	Hartley D, Moscovice I.	1996

### Outcomes:

The findings from the interview of the officials of the medical devices office at the Ministry of Health and Medical Education of Iran (MHME), and the review of the HTA study on the CT and MRI indicated that there was no difference between the mobile and the fixed CT and MRI scanners regarding the technical outcomes such as the diagnostic accuracy. This could be because the mobile MRI and CT scanners dumped into the trailer, are exactly the same as scanners which purchased for installation in a fixed room. In addition, other outcomes including the reduction staff workload (10), reduction in the time of receiving the diagnostic services (11, 18), enhancing patients’ recovery, reduction in the length of hospital stay, more accurate patient management, increased access and responsiveness to the patients (11, 19) were reported. The duration of receiving the diagnostic services results varied. Some studies reported a declining duration (12, 20) while others reported an increasing duration (21, 22). Since the eight studies reported different outcomes and because of the limited size of the eligible studies, meta-analysis was not feasible.

Besides, the studies were included in systematic reviews, examined the different outcomes and those with similar outcomes were based on qualitative studies. In none of the studies odds ratio, relative risk, probability, etc. were reported. Thus, the overall interpretation of the outcomes and safety of the scanners was based on the qualitative outcomes and expert opinions.

### Costs:

The estimated costs of the fixed and mobile CT scanner, as well as the purchasing and establishing costs of the fixed CT scanner, ranged from 524,182–757,742 $ (Table 4). The operating cost (provision, maintenance, staff, etc.) was about 122,082–141,930$ (assuming 25 patients per day). The fixed costs related to the purchasing and operating of the mobile CT scanners were in the range of 719,096–917,576$. The annual administrative costs were estimated at 141,131–161,017 $ (assuming 25 patients per day).

**Table 4:** Fixed and mobile CT Scan costs

***Costs***	***Fixed CT Scan***	***Mobile CT Scan***
CT Scanner (16 Tesla)	416,808 – 615,288	416,808 – 615,288
Wagon	N/A	4,857
Trailer	N/A	148,571
Siting and Energy Costs	N/A	148,860
Building	28,809 – 43,214	N/A
Concrete Pad	78,565 – 99,240	N/A
Total fixed cost	524,182 – 757,742	719,096 – 917,576
Facilities Maintenance and Equipment Upgrades	6,297	18,379
Staff	3,017	3,017
Operating Supplies special (tube)	63,142 – 82,990	63,142 – 82,990
common	49,626	49,626
Van Driver	N/A	6,857
Gasoline		110 – 148
Total variable cost	122,082 – 141,930	141,131 – 161,017
Total cost	646,264 – 899,672	860,227 – 1,078,593
Average cost	772,968	969,410

The fixed costs related to purchasing and establishing of fixed MRI was about 1,099,774–2,127,254 $ and the administrative cost was around 76,054 dollars (Table 5). The fixed costs of mobile MRI was in the range of 1,294,688–2,287,088 $ and the variable cost was about 99,713 – 99,751$.

**Table 5:** Fixed and mobile MRI costs

***Costs***	***Fixed MRI***	***Mobile MRI***
MRI Scanner (1/5 Tesla)	992,400 – 1,984,800	992,400 – 1,984,800
Wagon	N/A	4,857
Trailer	N/A	148,571
Siting and Energy Costs	N/A	148,860
Building	28,809 – 43,214	N/A
Concrete Pad	78,565 – 99,240	N/A
Total fixed cost	1,099,774 – 2,127,254	1,294,688 – 2,287,088
Facilities Maintenance and Equipment Upgrades	6,616	23,308
Staff	4,194	4,194
Operating Supplies special (Helium)	15,618	15,618
common	49,626	49,626
Van Driver	N/A	6,857
Gasoline		110 – 148
Total variable cost	76,054	99,713 – 99,751
Total cost	1,175,828 – 2,203,308	1,394,401 – 2,386,839
Average cost	1,689,568	1,890,620

### Economic evaluation:

Based on the findings from the interviews and review of the selected studies, especially the HTAs, technically the mobile MRI and CT scanners had same use as to that of the fixed ones. The cost-minimization analysis showed that the total cost of the CT scan in the first scenario was about 1,093,310$. The overall costs in the second were 2,368,144$, while in the third scenarios it was 557,614$. Among the three scenarios, the third scenario for purchasing and establishing one mobile CT scanner by all three cities that lacks fixed one had the lowest cost. The total costs of the MRI in the first, second and third scenarios were 3,130,951$, 8,688,109$ and 1,091,235$ respectively. In three hypothetical scenarios for MRI, the third scenario which is buying a mobile MRI scanner by the three hospitals had the lowest cost.

### Safety:

The CT-scanning is generally a noninvasive procedure. However, the radiation (x-ray) from this technology has the potential to cause risk to a fetus. About the safety of scanners it can be said that since there is no need for the invasive procedure in CT-scan, it is considered as a useful device to diagnose, but, because of the exposition of patients to X-Ray, paying attention to safe dose is essential. As there is some risk for the fetus in pregnant women, so CT-scan should be prescribed by a doctor. Generally, children are more vulnerable to x-ray than adults. Thus, CT-Scanners should be used only in emergency situations in urgent cases (7). The MRI, like CT-scan, is a non-invasive procedure. However, safety regulations should be taken into account in using it. Since its powerful magnetic field can pull steel bodies toward itself, it can create a danger to patients and to those in the path of such field. The patients who will undergo MRI test should not metal with them. A patient who has a metallic or magnetic implant or such as these in their tissues must inform to the radiologist before obtaining the imaging. Some MRI related tests might require injecting of a contrast material such as gadolinium into the bloodstream of the patient. The contrast does not have iodine and does not create sensitivity or other problems, whilst patients with the history of kidney disease, kidney-transplant, and liver disease should inform the radiologist before they undergo the imaging. Unlike the CT scanner, risks from the MRI on pregnant women have not been reported. However, the use of MRI should be limited to an essential item and the physician’s prescription (15). Prior to supplying the technology, the food and Drug Administration (FDA) confirmed that the efficacy of the mobile CT-Scan and MRI devices is similar to fixed ones (14). Since some patients may be concerned with closed spaces of mobile devices, such problems to be overcome by the presence of patient’s accompaniment or consulting with them. The trailers without changing room, a bridge should be embedded into a hospital for the convenience of patients. Generally, the safety of CT-Scan and MRI machines can be ensured because no complications have been reported for their users.

### Sensitivity analysis:

Like cost-effectiveness studies, it was not necessary to conduct sensitivity analysis because sensitivity analysis of the costs of the fixed and mobile CT-scan and MRI technologies were only based on the costs which were common among mobile and fixed devices and were changing in a given range. The cost tables for the CT-scan and MRI devices showed no difference between the fixed and variable costs of these technologies. Then, there was no given cost variable to impact on total costs and the selected scenario.

## Discussion

Buying a mobile CT scan or MRI for hospitals could be economically affordable if purchasing fixed devices is not cost-effective because of the low volume of patients. There is evidence showing the mobile devices would result in cost saving of 385.60 $ per scan (8). Although the mobile and fixed CT-Scan and MRI devices are technologically similar, this study identified some pros and cons of the mobile CT-Scan and MRI machines.

The mobile devices have lower cost of initial investment due to the cost sharing between several hospitals, and they required less time to set-up. In contrast, the fixed MRI or CT-Scanners needed the building of a unit or department. The mobile MRI and CT-Scanners can be useful when there is increased demand for scanning, and they can meet the hospitals demand in a short-term. However, in the long-term such advantages will be lost. From the perspective of patients, some of the advantages of mobile CT-Scan or MRI are time and cost saving. One of the disadvantages of mobile devices is the difficulty of coordination between hospitals, for example, if two or more hospitals are faced with demand surplus simultaneously, coordination between them will be very challenging. Additionally, devices are seldom close to hospitals so there would be no accessibility in emergency situations.

Recruitment of expert staff for working in mobile CT-Scan or MRI unit can be difficult and in some cases may be non-affordable. In addition, the operating cost of mobile CT-Scan or MRI is higher than the fixed ones and logistical needs can be problematic. From the patient’s point of view, one of the potential disadvantages of mobile CT-Scan or MRI is the possibility of increasing claustrophobia. The prevalence of the fear of enclosed area in mobile machines and the fixed ones is more than 10% and 1–4%, respectively (8). In a report in Belgium, mobile devices were used only to a right place is built in hospitals for fixed technologies (7). Barry et al.in their study reported the benefits of mobile MRI as providing services in areas where there is no access to MRI, maximizing the funding of service, improving in healthcare, focusing on selective outpatients, and support the services concerning neurology and orthopedics. On the other hand, they remarked lack of accessibility to MRI if it would be in another area, being inappropriate for complex scanning because of limited imagining space, as disadvantages (23). Finally by growth in access to mobile technology the amount of utilization may be increased.

### Limitations:

The systematic reviews and Health Technology Assessment (HTA) studies were rare. Hence, randomized controlled trial (RCT) and controlled clinical trial (CCT) studies were included in the study.

## Conclusion

Decision of policy makers and managers on purchasing a mobile CT-scan or MRI must not depend on the price solely; rather, they have to pay attention to all aspects like capacity of hospitals located near to each other for utilizing devices, population growth, pattern of diseases in the future and prices. Although the operating costs of mobile technology may be increased, these costs can be offset by the increasing in the visited patients, prorating of costs, flexibility, and other costs (in particular indirect Costs of patients). At the same time, most literature proposes the mobile MRI and CT-Scan as a provisional solution until setting up the fixed ones.

Therefore, from efficiency aspect, the basic outcomes that lead to overweight mobile MRI and CT-Scan to fixed one are the rise in patient’s access to diagnostic services, acceleration in patient’s management, and decrease in staff workload, moreover according to economic evaluation (cost-minimization), purchasing and utilization of a common mobile CT-scan or MRI by three or more hospitals have priority over that of fixed ones. The only difference between the safety of mobile and fixed devices described above is the fear of enclosed space in portable devices that can be solved by consulting with patients and the presence of patient’s accompaniment in the scanning room. Therefore, policymakers can accept decisions about purchasing mobile technology if buying a fixed technology do not have economic justification and hospitals are located in an appropriate distance from each other, otherwise, patient shortcoming can lead to induced demand by investors.

## Ethical considerations

Ethical issues (Including plagiarism, informed consent, misconduct, data fabrication and/or falsification, double publication and/or submission, redundancy, etc.) have been completely observed by the authors.

## References

[1] Murtagh J Warburton RN Foerster V Lentle BC Wood RJ Mensinkai S Husereau D (2006). CT and MRI for selected clinical disorders: a systematic review of economic evaluations [Technology report no 68]. Ottawa: Canadian Agency for Drugs and Technologies in Health.

[2] Westbrook C Talbot J (2018). MRI in practice. John Wiley & Sons.

[3] [No authors listed] (1987). Technology on wheels: Evaluating the options. Health Technol, 1(6):231–8. 10285677

[4] Fayad ZA Fuster V Nikolaou K Becker C (2002). Computed tomography and magnetic resonance imaging for noninvasive coronary angiography and plaque imaging: current and potential future concepts. Circulation, 106(15):2026–34. 12370230 10.1161/01.cir.0000034392.34211.fc

[5] National Imaging Associates, Inc (2016). NIA Clinical Guidelines for Medical Necessity Review. Magellan Healthcare,1–659.

[6] Demaerel P HR Verstraete K Bogaert J (2006). Magnetic Resonance Imaging Health Technology Assessment (HTA). Brussels: Belgian Health Care Knowledge Centre (KCE).

[7] Reeve J Baladi JF (1995). A comparison of fixed and mobile CT and MRI scanners. . Canadian Coordinating Office for Health Technology Assessment. National Library of Canada.

[8] Canadian Coordinating Office for Health Technology Assessment (1994). Selected health technologies in Canada (Technology brief; Issue 5.3). Ottawa: Canadian Coordinating Office for Health Technology Assessment.

[9] Butler WE Piaggio CM Constantinou C (1998). A mobile computed tomographic scanner with intraoperative and intensive care unit applications. Neurosurgery, 42(6):1304–10. 9632189 10.1097/00006123-199806000-00064

[10] Ebinger M Fiebach JB Audebert HJ (2015). Mobile computed tomography: prehospital diagnosis and treatment of stroke. Curr Opin Neurol, 28(1):4–9. 25490196 10.1097/WCO.0000000000000165

[11] Gunnarsson T Theodorsson A Karlsson P (2000). Mobile computerized tomography scanning in the neurosurgery intensive care unit: increase in patient safety and reduction of staff workload. J Neurosurg, 93(3):432–6. 10969941 10.3171/jns.2000.93.3.0432

[12] Walter S Kostopoulos P Haass A (2012). Diagnosis and treatment of patients with stroke in a mobile stroke unit versus in hospital: a randomised controlled trial. Lancet Neurol, 11(5):397–404. 22497929 10.1016/S1474-4422(12)70057-1

[13] Boulevard J B (2013). Patient Safety: Magnetic Resonance Imaging (MRI). Radiological Society of North America.

[14] Mansori M What is CT Scan and how is it work? (2012) IRAN: Iran ortoped; Available from: http://www.iranorthoped.ir/fa/news/1357

[15] Kieso DE Weygandt JJ Warfield TD (2010). Intermediate accounting: IFRS edition. 3rd ed John Wiley & Sons, USA, pp.: 201–305.

[16] [No authors listed] (2011). Law for the Fifth Development Plan of the Islamic Republic of Iran: https://wipolex.wipo.int/en/legislation/details/14565

[17] Shawi M Al Watson T Carlson J Anthony L Thomas M (2013). CT Scan Services In The Rural Setting, The Clinical Need And Cost Effectiveness: The Katherine Hospital Experience (Australia). The Internet Journal of Surgery, 30(1):1–4.

[18] Tsang K I Cheung TI Liu H (2009). Mobile Computed Tomography Scanner for Head and Neck Imaging. Hong Kong: society of critical care medicine.

[19] Matson M Jarosz J Gallacher D (1999). Evaluation of head examinations produced with a mobile CT unit. Br J Radiol, 72(859):631–6. 10624318 10.1259/bjr.72.859.10624318

[20] Hartley D Moscovice I (1996). The mobile hospital technology industry: focus on the computerized tomography scanner. J Rural Health, 12(3):225–34. 10162854 10.1111/j.1748-0361.1996.tb00797.x

[21] Teichgräber UK Pinkernelle J Jürgensen J-S Ricke J Kaisers U (2003). Portable computed tomography performed on the intensive care unit. Intensive Care Med, 29(3):491–5. 12545344 10.1007/s00134-002-1606-x

[22] Adelman AM Daly MP (2005). Initial evaluation of the patient with suspected dementia. Am Fam Physician, 71(9):1745–50. 15887453

[23] Rumboldt Z Huda W (2009). Review of Portable CT with Assessment of a Dedicated Head CT Scanner. AJNR Am J Neuroradiol, 30(9):1630–6. 19661166 10.3174/ajnr.A1603 PMC7051518

